# CircRIC8B regulates the lipid metabolism of chronic lymphocytic leukemia through miR199b-5p/LPL axis

**DOI:** 10.1186/s40164-022-00302-0

**Published:** 2022-09-05

**Authors:** Zijuan Wu, Danling Gu, Ruixin Wang, Xiaoling Zuo, Huayuan Zhu, Luqiao Wang, Xueying Lu, Yi Xia, Shuchao Qin, Wei Zhang, Wei Xu, Lei Fan, Jianyong Li, Hui Jin

**Affiliations:** 1 Department of Hematology, Pukou CLL Center, the First Affiliated Hospital of Nanjing Medical University, Jiangsu Province Hospital, Nanjing, 210029 China; 2 Key Laboratory of Hematology of Nanjing Medical University, Nanjing, 210029 China; 3 Jiangsu Key Lab of Cancer Biomarkers, Prevention and Treatment, Collaborative Innovation Center for Personalized Cancer Medicine, Nanjing Medical University, Nanjing, 210029 China; 4 National Clinical Research Center for Hematologic Diseases, the First Affiliated Hospital of Soochow University, Suzhou, 215000 China

**Keywords:** Chronic lymphocytic leukemia, Lipid metabolism, circRIC8B, LPL, Ezetimibe

## Abstract

**Objective:**

Circular RNAs (circRNAs) play a critical role in the modulation of tumor metabolism. However, the expression patterns and metabolic function of circRNAs in chronic lymphocytic leukemia (CLL) remain largely unknown. This study aimed to elucidate the role of circRNAs in the lipid metabolism of CLL.

**Methods:**

The expression and metabolic patterns of circRNAs in a cohort of 53 patients with CLL were investigated using whole transcriptome sequencing. Cell viability, liquid chromatography with tandem mass spectrometry (LC–MS/MS) analysis, lipid analysis, Nile red staining as well as triglyceride (TG) assay were used to evaluate the biological function of circRIC8B in CLL. The regulatory mechanisms of circRIC8B/miR-199b-5p/lipoprotein lipase (LPL) axis were explored by luciferase assay, RNA immunoprecipitation (RIP), qRT-PCR, and fluorescence in situ hybridization (FISH). CCK-8 and flow cytometry were used to verify the inhibition role of cholesterol absorption inhibitor, ezetimibe, in CLL cells.

**Results:**

Increased circRIC8B expression was positively correlated with advanced progression and poor prognosis. Knockdown of circRIC8B significantly suppressed the proliferation and lipid accumulation of CLL cells. In contrast, the upregulation of circRIC8B exerted opposite effects. Mechanistically, circRIC8B acted as a sponge of miR-199b-5p and prevented it from decreasing the level of LPL mRNA, and this promotes lipid metabolism alteration and facilitates the progression of CLL. What’s more, ezetimibe suppressed the expression of LPL mRNA and inhibited the growth of CLL cells.

**Conclusions:**

In this study, the expressional and metabolic patterns of circRNAs in CLL was illustrated for the 1st time. Our findings revealed that circRIC8B regulates the lipid metabolism abnormalities in and development of CLL through the miR-199b-5p/LPL axis. CircRIC8B may serve as a promising prognostic marker and therapeutic target, which enhances the sensitivity to ezetimibe in CLL.

**Supplementary Information:**

The online version contains supplementary material available at 10.1186/s40164-022-00302-0.

## Introduction

Chronic lymphocytic leukemia (CLL), which is particularly prevalent in Western countries, has shown an increasing incidence rate in China [1–3]. Characterized by marked clinical heterogeneity, approximately 1/3 of patients with CLL experience a rapidly progressive clinical course [4]. Despite extensive research and progress, therapies for CLL remain inadequate. Novel biomarkers for the prediction of CLL prognosis and further elucidation of the precise molecular mechanisms can improve the outcome of CLL patients.

Accumulating evidence has illustrated the pivotal role of metabolism in tumors [5–7], and metabolism pattern is dictated by a variety of intrinsic and extrinsic factors. Non-coding RNAs (ncRNAs), including circular RNAs (circRNAs) was reported to influence tumor metabolism [8]. For example, circACC1 contributes to metabolic adaptation and acts as a tumor promoter in colorectal cancer [9], and circRNA SCAR could regulate the metabolic inflammation of nonalcoholic steatohepatitis [10]. In order to satisfy the energy demand, CLL cells could store lipids and utilize free fatty acids (FFAs) [11]. Lipoprotein lipase (LPL) was found to be a key factor in the metabolism of CLL cells by promoting cellular uptake of lipoproteins and accelerating the hydrolysis of triglycerides (TGs) into FFAs. Ezetimibe, a FDA-approved drug could significantly reduce the levels of low-density lipoprotein cholesterol (LDL-C) and TGs by inhibiting intestinal absorption of cholesterol [12]. Numerous studies claimed that CLL patients with higher LPL levels were usually correlated with aggressive disease and unfavorable prognosis [13, 14]. However, the role of circRNAs in mediating the metabolism of CLL cells, particularly in lipid metabolism, has not yet been reported. Therefore, we attempted to identify the metabolism-associated circRNA profiles in CLL and explored the underlying molecular mechanisms.

Here, we identified circRIC8B, which promotes cell proliferation and lipid metabolism and is associated with poor outcomes in patients with CLL. Additionally, circRIC8B functions as an oncogenic driver to promote lipid metabolism by sponging miR-199b-5p, resulting in the upregulation of LPL mRNA. For the 1st time, our study explored the relationship between circRNAs and metabolism in CLL. These findings also provide new evidence that circRNAs function in CLL and offer a promising therapeutic target, broadening treatment options that targeting metabolism.

## Materials and methods

### Human samples

Peripheral blood samples were obtained from previously untreated patients with CLL who were followed up at the First Affiliated Hospital of Nanjing Medical University, Jiangsu Province Hospital. Written informed consent was obtained from the patients and use of human samples was approved by the ethics committee (Approval Number 2021-SRFA-208). Peripheral blood mononuclear cells (PBMCs) were isolated using Lymphoprep™ (Stemcell Technologies, Vancouver, Canada) and stored in TRIzol at− 80 °C until use.

### Cell culture

CLL cell lines MEC-1 and JVM-3 (X–Y Biotechnology, Shanghai, China) and Human B lymphocyte cell line GM12878 were cultured in Roswell Park Memorial Institute (RPMI) 1640 medium (Gibco, Grand Island, USA) supplemented with 10% fetal bovine serum (FBS) (Yeasen, Shanghai, China) and 100 μg/mL penicillin/streptomycin (Gibco). Human renal epithelial carcinoma HEK293T cells were cultured in Dulbecco’s modified Eagle’s medium (DMEM) (Gibco). The cell lines used in this study were authenticated by short tandem repeat (STR) profiling and maintained at 37 °C in a humidified atmosphere with 5% CO_2_.

### Cell transfection

Transient transfection with small interfering RNAs (siRNAs) specifically targeting circRIC8B that synthesized by Geenseed Biotech (Guangzhou, China) was performed by using Lipofectamine^®^ RNAiMAX Transfection Reagent (Invitrogen) according to the manufacturer’s protocol. Briefly, six thousand cells washed twice with sterile PBS were seeded in a 6-well plate. 100 nM siRNAs and the same volume of RNAiMAX were diluted with OPTI-MEM medium separately and mixed and incubated for 20 min before added into cells. For transient transfection, cells are typically assayed 48 h after transfection. To generate CLL cell clines that stably overexpress circRIC8B, MEC-1 and JVM-3 were infected with lentivirus vectors (Geenseed Biotech) according to the manufacturer’s protocol. After 3 days of infection, cells were selected with puromycin (2 µg/ml) for 3 days.

### RNA extraction and quantitative real-time PCR

Total RNA was extracted using TRIzol reagent according to the manufacturer’s protocol. For RNase R treatment, 2.5 μg of total RNA was incubated with or without 3 U/μg of RNase R for 20 min at 37 °C (Epicentre Technologies, Madison, WI). cDNA was synthesized using a reverse transcription kit (Vazyme, Nanjing, China). For actinomycin D treatment, the culture medium was added with actinomycin D (2 ug/mL) and cells were collected at a specified time to assess the stability of circRNA. The separation of nuclear and cytoplasmic fractions of cells were performed using PARIS™ Kit (Life Technologies). qRT-PCR was performed with SYBR Green Master Mix (Vazyme, Nanjing, China). The specific primers used are listed in Additional file 1: Table S1. GAPDH or U6 was used as an internal standard and relative RNA expression levels were calculated using the 2^–ΔΔCT^ method (See Additional file 1: Table S1).

### Cell proliferation and growth inhibition assay

For cell proliferation analysis, Cell Counting Kit-8 (CCK-8; APExBIO, Houston, TX, USA) was used. Briefly, 10 000 cells were seeded in 96-well plates and ten microliters of CCK-8 assay solution were added to each well at the indicated times. After 2 h’s incubation, absorbance at 450 nm was measured. For growth inhibition analysis, cells were incubated with the indicated concentrations of ezetimibe (Selleck Chemicals, S1655) range from 0 to 40 μM for 48 h.

### Flow cytometry

For the cell apoptosis assay, cells were firstly washed twice with cold PBS and resuspended with binding buffer. Apoptotic levels of cells were quantified by flow cytometry (BD Biosciences) after 15 min’s incubation at 4 °C with Annexin V and PI. Data analysis was performed using the CytExpert software.

### Fluorescence in situ hybridization (FISH)

The FISH assay was performed to observe the location of circRIC8B and miR-199b-5p in CLL cells. Cy3-labeled circRIC8B probes and 5FAM-labeled miR-199b-5p were designed and synthesized by RiboBio (Guangzhou, China). FISH analysis was performed using a fluorescent in situ hybridization kit (RiboBio) according to the manufacturer’s instructions. Cell nuclei were stained with 4,6-diamidino-2-phenylindole (DAPI; Beyotime, China). Images were obtained using a fluorescence microscope.

### RNA immunoprecipitation (RIP)

The RIP assay was performed using the Magna RIP™ RNA-binding protein immunoprecipitation kit (Millipore, USA) following the manufacturer’s protocol. Briefly, 6 × 10^7^ cells were collected and resuspended in 300 μL complete RIP lysis buffer. The cell lysates were incubated with antibodies against Argonaute-2 (AGO2; Abcam, MA, USA) or rabbit IgG and pre-treated magnetic beads at 4 °C overnight with rotation. Immunoprecipitated RNA was then reverse transcripted after purification. The enrichment of circRIC8B was evaluated using qRT-PCR.

### Nile red staining and triglyceride (TG) assay

Intracellular lipid droplet analysis was performed using Nile red staining. Cells were treated with 1 mM FFA and fixed with 4% paraformaldehyde for 15 min. After staining with Nile red dye (1 μg/mL), the images were acquired using a fluorescence microscope. TG content in CLL cells was quantified using a triglyceride quantification kit (BioAssay Systems, ETGA-200) according to the manufacturer's instructions.

### Dual-Luciferase reporter assay

HEK293 T cells were seeded in 24-well plates at 30% confluency for 24 h before transfection. The cells were co-transfected with a mixture of luciferase reporter vectors (pmirGLO) containing circRIC8B-miRNA binding or mutant sequences and miRNA mimics to examine their miRNA binding ability. After 48 h, luciferase activity was measured using a dual-luciferase reporter assay system (Promega, Madison, WI, USA) according to the manufacturer’s protocol and a previous study [15].

### Whole transcriptome sequencing

Total RNA was extracted from patients with CLL, followed by rRNA depletion. Then, the RNA was reverse transcribed to cDNA and constructed into a strand-specific library after purification by rRNA depletion. Sequencing was performed on an Illumina Novaseq 6000 platform (Illumina, San Diego, CA, USA). All raw data is accessible through the NCBI GEO database (SRA: PRJNA762572). GSVA was used to obtain the metabolic score and the subsequent metabolic heatmap [16].

### Liquid chromatography with tandem mass spectrometry (LC–MS/MS) analyses

A total of 10^7^ cells were collected and washed twice with PBS. For metabolite extraction, an extraction solution (acetonitrile: methanol: water = 2: 2: 1) containing an isotopically labeled internal standard mixture was added to the sample. After a 30 s vortex, the samples were frozen and thawed with liquid nitrogen 3 times and then sonicated for 10 min in an ice-water bath. Supernatant was transferred to a fresh glass vial for LC–MS/MS analysis after centrifugation. Quality control (QC) samples were prepared by mixing an equal aliquot of the supernatant from all samples. LC–MS/MS analyses were performed using a UHPLC system (Vanquish, Thermo Fisher Scientific). Raw data were converted to the mzXML format using ProteoWizard and processed with an in-house program, developed using R and based on XCMS, for peak detection, extraction, alignment, and integration. An in-house MS2 database (BiotreeDB) was then used for metabolite annotation. The cutoff for annotation was set at 0.3. Detection and analysis were provided by SHANGHAI BIOTREE BIOTECH CO., LTD.

### Statistical analyses

Graphpad Prism software version 8 was used to statistically analyze the data. All of the experiments were conducted in triplicate. P values were calculated using a Student’s t-test. Spearman’s rank correlation coefficient with t-test was used to evaluate correlative studies. Time to first treatment (TTT) was calculated from the date of diagnosis to the date of first treatment or last follow-up, considering disease-unrelated deaths as competing events. The Kaplan–Meier method and log-rank test were used to assess overall survival, and the Gray test was used to compare the cumulative incidence curves of TTT. Multivariate analyses of prognostic factors were performed using the Cox regression models. Statistical significance was set at *p* < 0.05.

## Results

### Properties of circRNAs in CLL

**Fig. 1:**
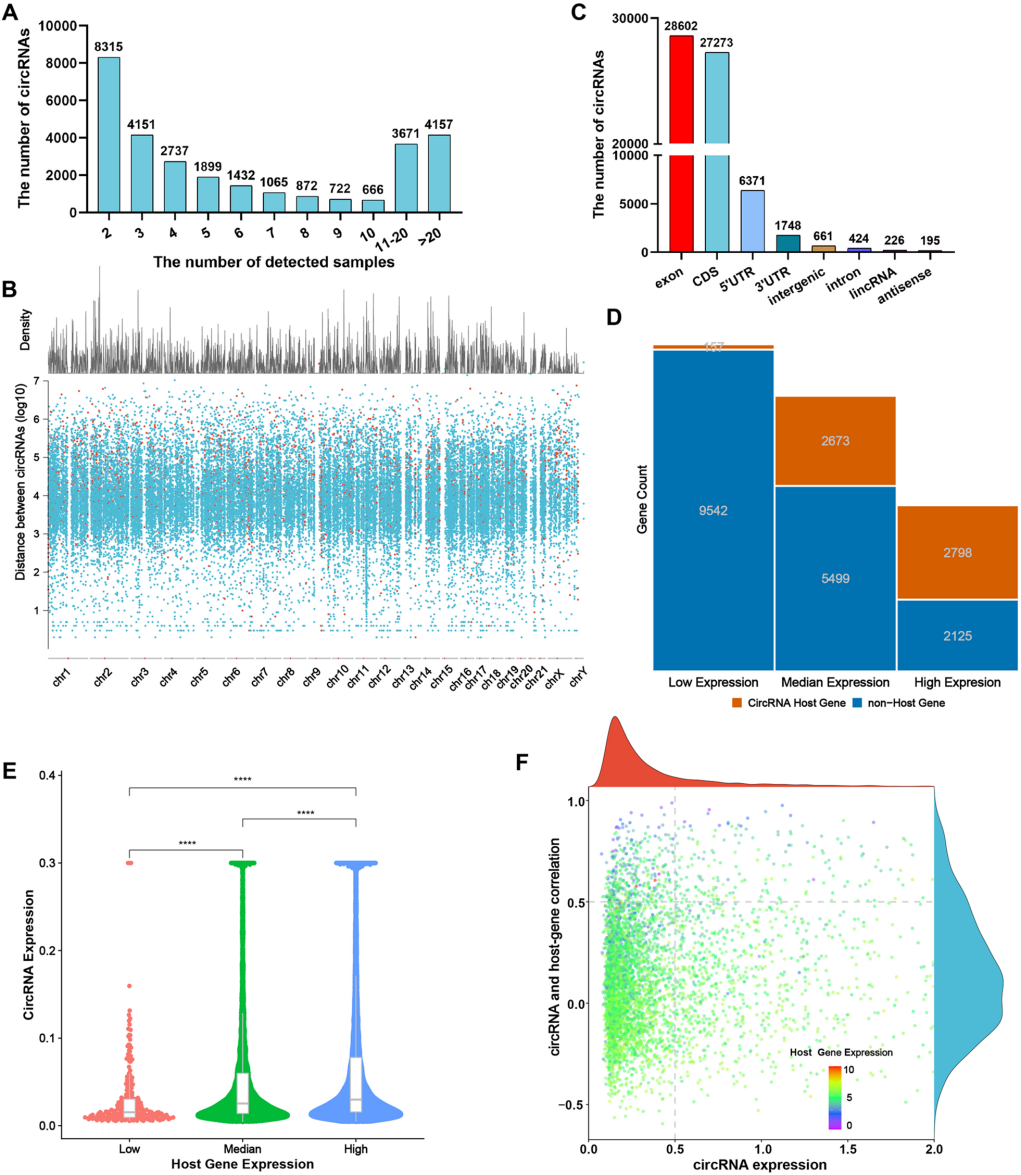
Profiling of circular RNAs in PBMCs from CLL patients. **A** The number of circRNAs identified in PBMCs from patients with CLL (n = 53). **B** Genomic origin of human circRNAs. **C** CircRNA rainfall plot for circRNAs in different chromosomes. **D** Count of circRNAs generated from different expression levels of host genes. **E** Expression level of circRNA in three groups of genes. **F** Correlation between circRNA abundance and linear host-gene expression

In our previous study, we elucidated the expression profile of circRNAs in the plasma of patients with CLL [15]. To further explore the profile of cells from CLL patients, 53 treatment-naïve patients with CLL were enrolled. Whole transcriptome sequencing showed that circRNAs were broadly expressed and at least 4000 circRNAs were captured in the majority of samples (Fig. 1A). These circRNAs were found generated from various genomic regions, including untranslated regions (UTRs), intergenic regions, introns, and most commonly protein-coding exons (Fig. 1B). In addition, the density distribution of circRNAs on different chromosomes are displayed in Fig. 1C. As most circRNAs are emanated from protein-coding genes, we then wondered the relationship between the abundance of circRNAs and their host genes. Host genes with higher mRNA levels tend to transcribe circRNAs (Fig. 1D). To further elucidate the correlation between host genes and circRNAs, host genes were divided into three groups according to their abundance. With a higher expression of host genes, circRNAs were likely to exhibit higher abundance (Fig. 1E). Moreover, our results indicated that the host genes with lower abundance of mRNAs showed a stronger positive correlation with the expression of their corresponding circRNAs (Fig. 1F). Collectively, these results illustrated that circRNAs are highly and broadly expressed in CLL patients.

### CircRNAs involved in the metabolism of CLL

**Fig. 2:**
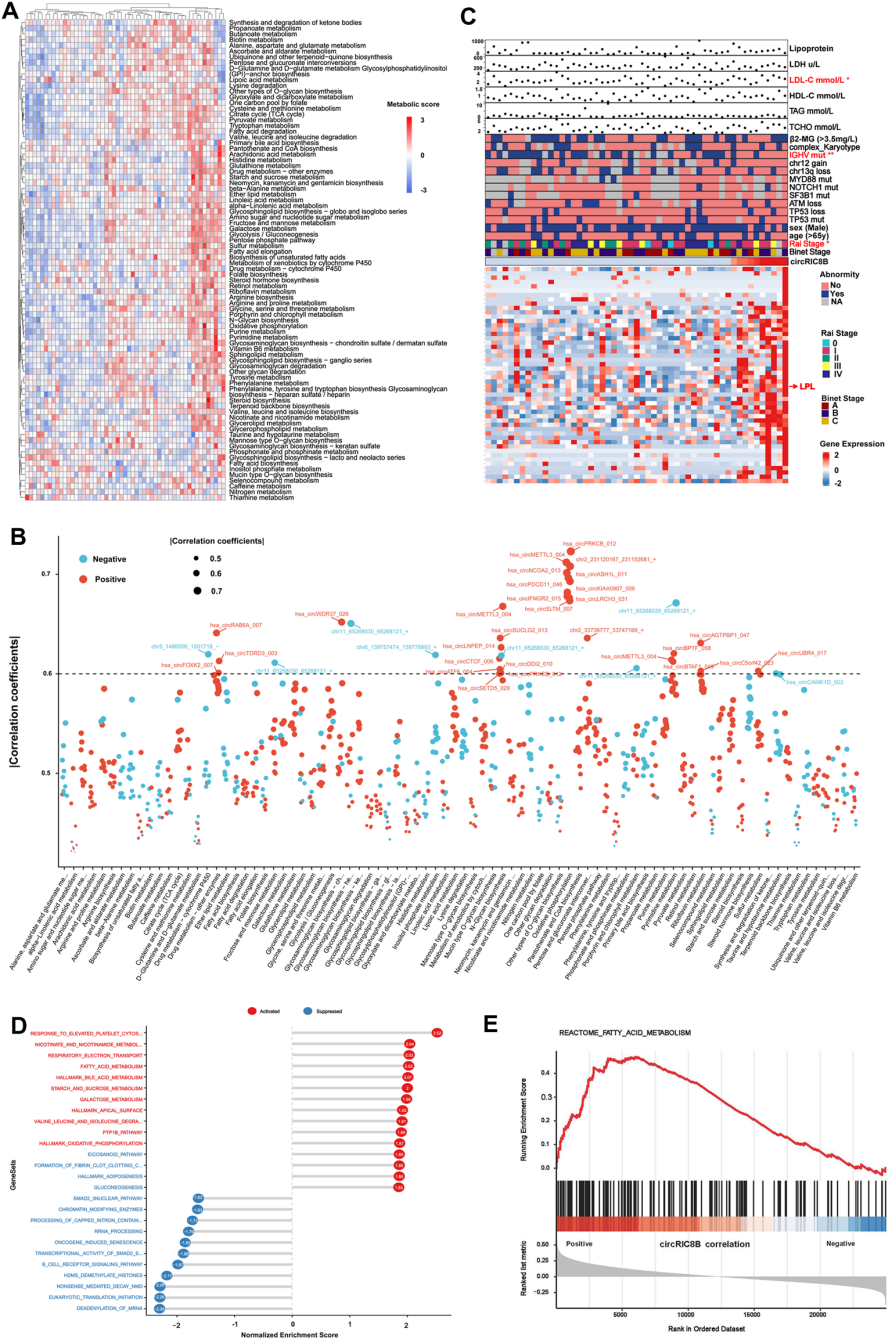
Metabolic levels in patients with CLL and related circRNAs. **A** Heatmap of CLL patients with different metabolic levels. **B** circRNAs that correlated with metabolic pathways. **C** Above: Univariate analysis identified factors corresponding to the expression of circRIC8B. Below: Genes that positively correlated with circRIC8B. **D**, **E** Gene set enrichment analysis of circRIC8B-related genes and circRIC8B expression was significantly correlated with fatty acid metabolism. ^*^P < 0.05, ^**^P < 0.01, ^***^P < 0.001

To mine the circRNAs that regulating metabolism, the scores of various metabolic pathways in patients with CLL were firstly calculated using the mRNA sequencing data (Additioinal file 2: Table S2). In addition, the heatmap exhibited the enrichment of metabolism pathways of individual patient (Fig. 2A), and relevant circRNAs were shown in Fig. 2B. Given the significance of LPL in lipid metabolism, circRIC8B with proper length and relative high abundance, among circRNAs that positively correlated with LPL was selected for further study. Participant characteristics were collected and summarized (Fig. 2C). The expression levels of circRIC8B were observed significantly correlated with CLL patients’ elevated low-density lipoprotein cholesterol (LDL-C) levels. In addition, patients with higher circRIC8B levels were accompanied with more, advanced Rai stage, and unmutated immunoglobulin heavy-chain variable region (IGHV) status. Meanwhile, LPL was observed positively associated with circRIC8B expression (Additional file 3: Table S3). Gene set enrichment analysis (GESA) of associated genes was also performed, and the results revealed that circRIC8B was involved in metabolism, including fatty acid metabolism (Fig. 2D, E).

### Characterization of circRIC8B in CLL

**Fig. 3:**
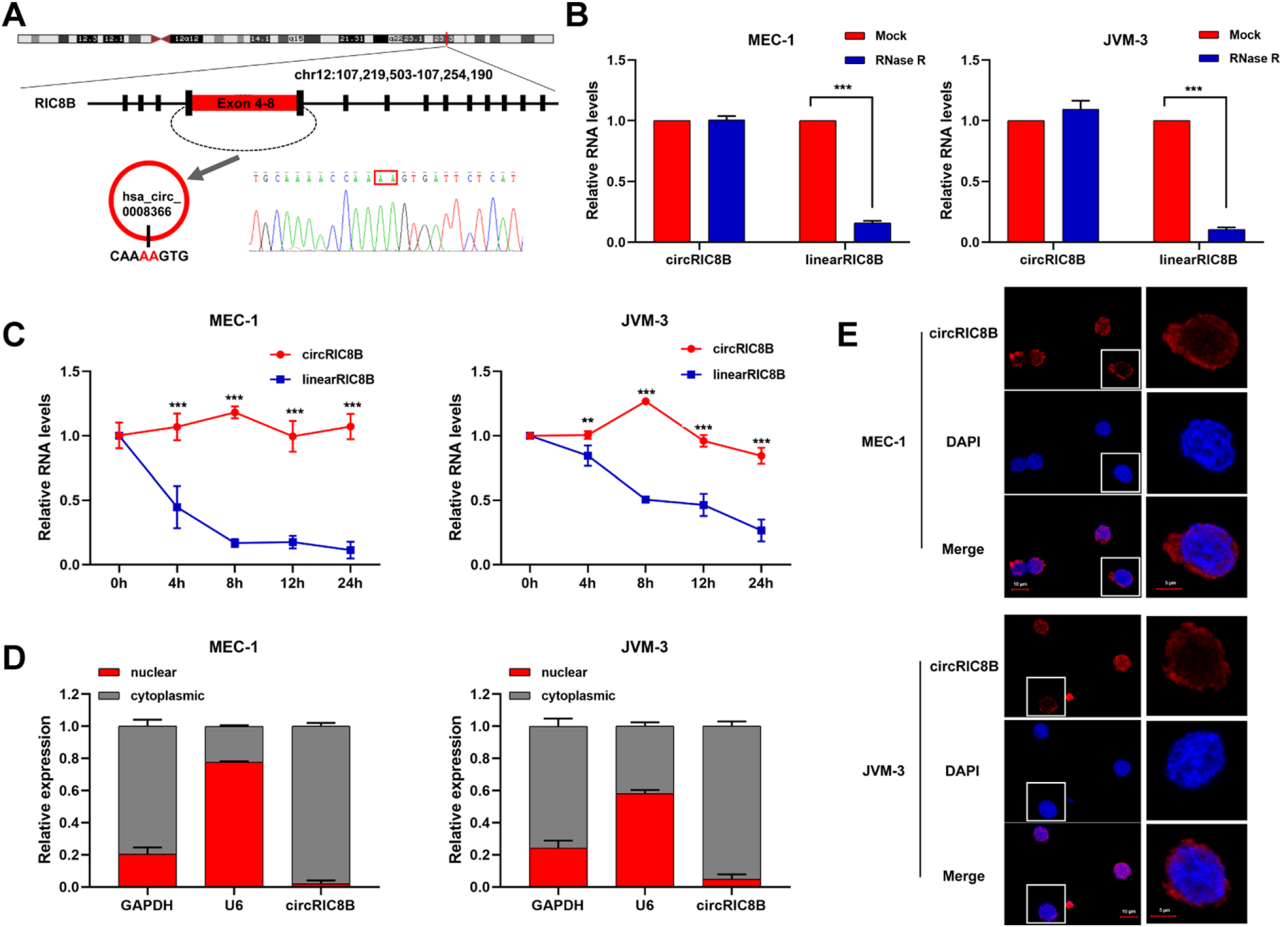
The characteristics of circRIC8B. **A** The annotated region in RIC8B gene for the formation of circRIC8B was shown. The exact sequence of the back splicing site in circRIC8B was confirmed by sequencing. **B** qRT–PCR for the abundance of circRIC8B and RIC8B mRNA in CLL cells treated with RNase R. Data are shown as means ± SD (n = 3, ^***^P < 0.001). **C** qRT–PCR for the abundance of circRIC8B and RIC8B mRNA in CLL cells treated with actinomycin D at the indicated time points. Data are shown as means ± SD (n = 3, ^**^P < 0.01, ^***^P < 0.0001). **D** Nuclear and cytoplasmic fraction assay for the determination of the location of circRIC8B and RIC8B in CLL cells. U6 were applied as positive controls. **E** RNA-FISH for circRIC8B. circRIC8B was stained with cy3 labeled probe. Nuclei were stained with DAPI. Scale bar, 10 μm. Data are shown as means ± SD (n = 3, ^**^P < 0.01, ^***^P < 0.001)

circRIC8B was generated from the head-to-tail splicing of exons 4 to 8 from RIC8B precursor-mRNA (pre-mRNA) (710 bp). Primers were designed according to the back-splicing junction and the head-to-tail splicing was confirmed with sanger sequencing (Fig. 3A). The RNase R digestion assay demonstrated that circRIC8B was resistant to RNase R, whereas the linear isoform was decreased after RNase R treatment (Fig. 3B). In addition, circRIC8B was more stable than RIC8B mRNA in CLL cells treated with actinomycin D, a transcription inhibitor (Fig. 3C). Nuclear and cytoplasmic fraction assays and fluorescence in situ hybridization (FISH) showed circRIC8B was predominantly distributed in cytoplasm (Fig. 3D, E). Collectively, these findings demonstrated that circRIC8B was an abundant, circular, and stable transcript that was expressed in CLL cells.

### CircRIC8B predicts aggressive clinical characteristics in patients with CLL and promotes cell proliferation

**Fig. 4:**
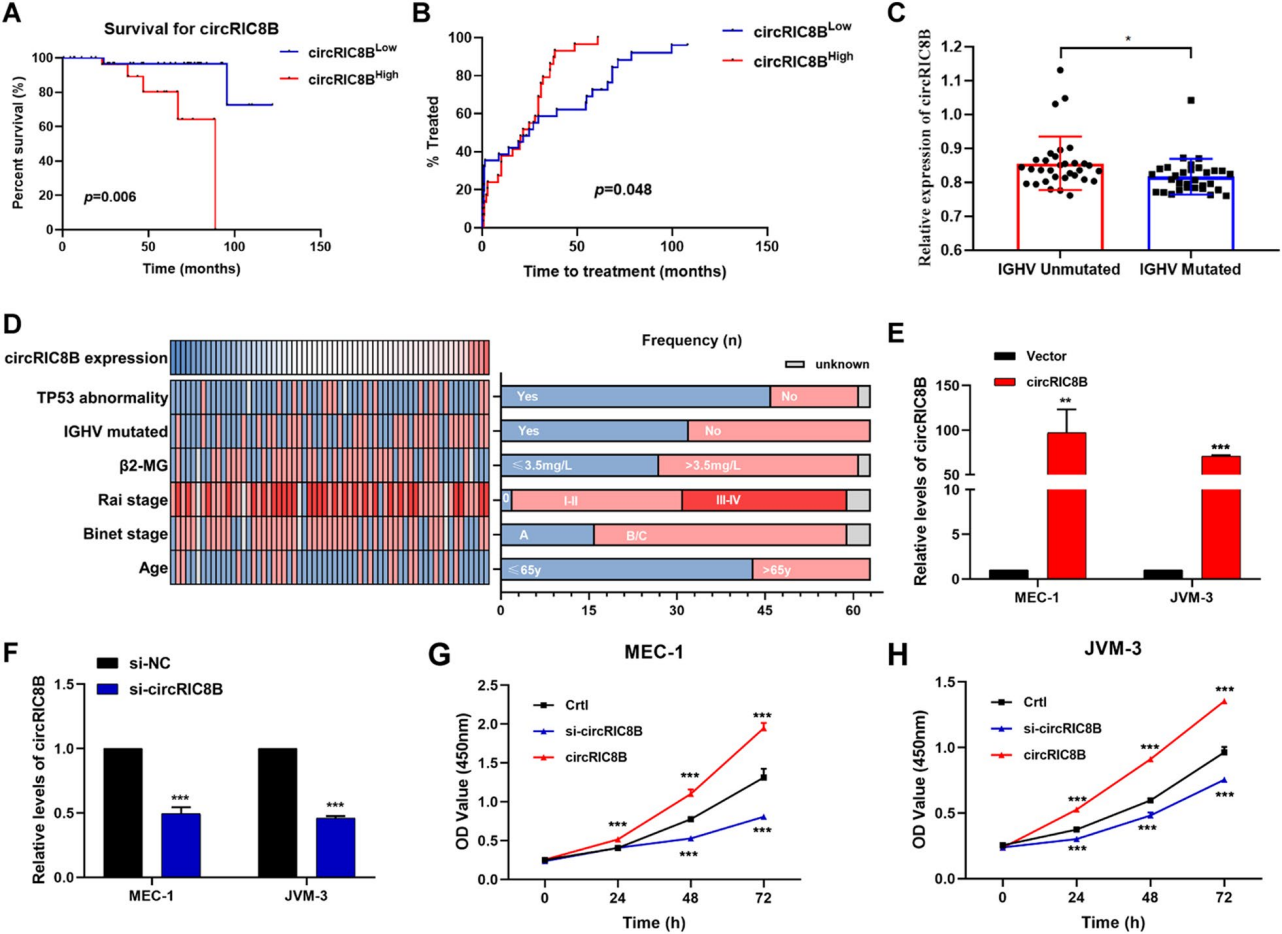
Clinical significance of biological functions of circRIC8B. **A** Kaplan–Meier plot analysed the association between circRIC8B expression level and overall survival. Log-rank tests were used to determine statistical significance (n = 63). **B** Comparison of TTT among patients with high or low levels of circRIC8B. **C** Expression levels of circRIC8B in patients with or without IGHV mutation. **D** Clinical characteristics of patients with CLL enrolled in this group. **E**, **F** circRIC8B expression in CLL cells transfected with circRIC8B overexpression plasmids or siRNAs by qRT-PCR. **G**, **H** Growth curves of cells measured by CCK-8 assay. N = 3 (mean ± SD); ^*^P < 0.05, ^**^P < 0.01 and ^***^P < 0.001 by Student’s t‐test

Further PBMC samples from 63 CLL patients were collected to detect the expression levels of circRIC8B and confirm its clinical significance. Kaplan–Meier analysis displayed that patients with higher circRIC8B expression had a worse prognosis and shorter survival time (Fig. 4A). Moreover, patients with CLL who had low levels of circRIC8B had a notably longer time to first treatment time (TTT) (Fig. 4B). The results were the same as those for the LPL (Additional file 4: Fig S1A, B). In addition, the expression levels of circRIC8B and LPL mRNA were elevated in patients without IGHV mutation compared to those in the IGHV mutated patients (Fig. 4C, Additional file 4: Fig S1C). In line with the TCGA database [17], IGHV mutation status corresponded with the expression of LPL mRNA (Fig. 4C, Additional file 1: Fig S1D). The relationship between circRIC8B level and other clinicopathological characteristics was also evaluated (Fig. 4D). The expression of circRIC8B was found to be associated with IGHV mutation status rather than other clinical characteristics, including age, sex, cytogenetics, and stage (Table 1). To eliminate the possible contamination of other cells in PBMCs, CLL B cells from 10 CLL patients were isolated with CD19 MicroBead (Miltenyi Biotec, 130–050-301). circRIC8B expression of CD19 + and CD19- cells were separately detected. And the results showed that circRIC8B expression was significantly highly expressed in CLL-B cells (CD19 +) compared to other cells (Additional file 5: Fig S2A). What’ more, CLL cell lines MEC-1 and JVM-3 showed the relative higher levels compared to Human B lymphocyte cells GM12878 (Additional file 5: Fig S2B). We further investigated the effects of circRIC8B on cell proliferation and small interfering RNAs (siRNAs) that specifically target circRIC8B and circRIC8B overexpression vector were constructed and validated by agarose gel electrophoresis and sanger sequencing (Additional file 6: Fig S3A and B). The knockdown and overexpression efficacy were validated by qRT-RCR (Fig. 4E and F, Additional file 6: Fig S3C and D). Cell counting kit-8 proliferation (CCK-8) assay showed that knockdown of circRIC8B notably suppressed the proliferation ability of CLL cells, whereas overexpression of circRIC8B led to an increase in cell viability (Fig. 4E–H). These data suggested that circRIC8B plays an oncogenic role in CLL.

**Table 1:** Correlation between circRIC8B expression and clinical characteristics in 63 CLL patients

Characteristic	All cases	circRIC8B		Chi-square	*P* value
		low	high		
All cases	63	31	32		
Age					
≤ 65y	44	21	23	0.026	0.871
> 65y	18	9	9		
Sex					
Male	43	21	22	0.007	0.932
Female	20	10	10		
Rai stage					
0	2	1	1	0.587	0.444
I-II	29	12	17		
III-IV	28	15	13		
Binet stage					
A	27	13	14	0.021	0.886
B/C	34	17	17		
TP53 abnormality					
Absent	46	25	21	1.999	0.157
Present	15	5	10		
IGHV status					
Unmutated	30	11	19	5.670	0.017*
Mutated	33	22	11		
Trisomy 12					
Absent	42	19	23	0.003	0.954
Present	13	6	7		
Del(13q)					
Absent	30	16	14	1.652	0.199
Present	25	9	16		
ATM deletion					
Absent	46	22	24	0.020	0.887
Present	11	5	6		
Cytogenetics					
Diploid	35	14	21	1.586	0.208
Complex	19	11	8		
β2-MG					
≤ 3.5 mg/L	27	13	14	0.021	0.886
> 3.5 mg/L	34	17	17		

*IGHV* immunoglobulin heavy-chain variable region, *Del* deletion, *β2-MG* β2-microglobulin

^*^P < 0,05

### CircRIC8B regulates lipid metabolism in CLL

**Fig. 5:**
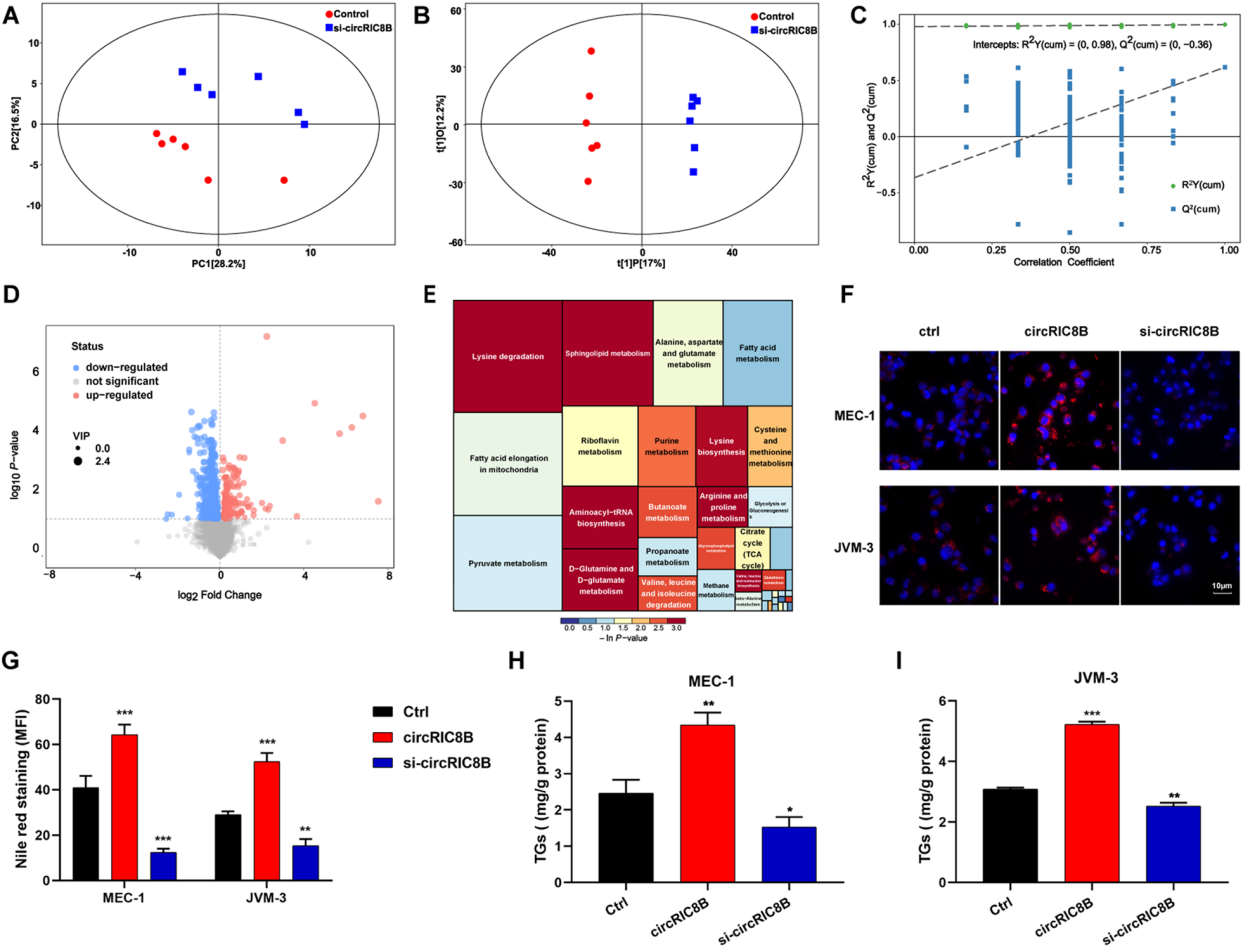
Clinical significance of biological functions of circRIC8B.** A** PCA (principal component analysis) score plot, **B** OPLS-DA (orthogonal projections to latent structures-discriminant analysis,) score plot, and **C** permutation test of OPLS-DA model between the control group and si-circRIC8B group. **D** Volcano plot presented the dysregulated metabolites. VIP, variable importance in the projection. **E** Metabolic pathway mapping of the impacted metabolic network. **F**, **G** Photomicrographs and quantitative analysis of Nile red staining. Scale bar, 10 μm. **H**, **I** Relative TG (mg/g protein) contents. TG, Triglyceride. Data are shown as means ± SD (n = 3, ^*^P < 0.05, ^**^P < 0.01, ^***^P < 0.001)

To determine the role of circRIC8B in metabolism, liquid chromatography with tandem mass spectrometry (LC–MS/MS) analysis was performed with six biological replicates between cells with or without circRIC8B siRNA interference. The principal component analysis (PCA) score plot showed a clear separation between the si-circRIC8B and control groups (Fig. 5A). The identified metabolites were then used to perform supervised clustering, orthogonal partial least squares discriminant analysis (OPLS-DA) (Fig. 5B). The analysis produced a strong model with highly validated predictability and goodness of fit (Fig. 5C). Volcano plots showed that the metabolites were differentially expressed between the two groups (Fig. 5D, Additional file 7: Table S4). Metabolic pathway analysis was performed, and the lipid metabolism pathway was observed the most considerably altered between the two groups (Fig. 5E). This indicated that circRIC8B was a potential regulator that plays a key role in lipid metabolism. To test this hypothesis, Nile red staining was performed (Fig. 5F), quantitative analysis of mean fluorescence intensity (MFI) was performed (Fig. 5G). The results showed that lipid accumulation was increased in CLL cell lines with circRIC8B overexpression, whereas a significant decrease in lipid accumulation was observed in the circRIC8B knockdown cells. Besides, cells overexpressing circRIC8B exhibited a higher Triglyceride (TG) content, which is a quantitative indicator of lipid accumulation, whereas a lower TG content was detected in the circRIC8B knockdown cells, in line with the results of Nile red staining. Taken together, circRIC8B was regarded as a key factor that regulates lipid accumulation and then promoted cell proliferation in CLL (Fig. 5H, I).

### CircRIC8B serves as a sponge of miR-199b-5p

**Fig. 6:**
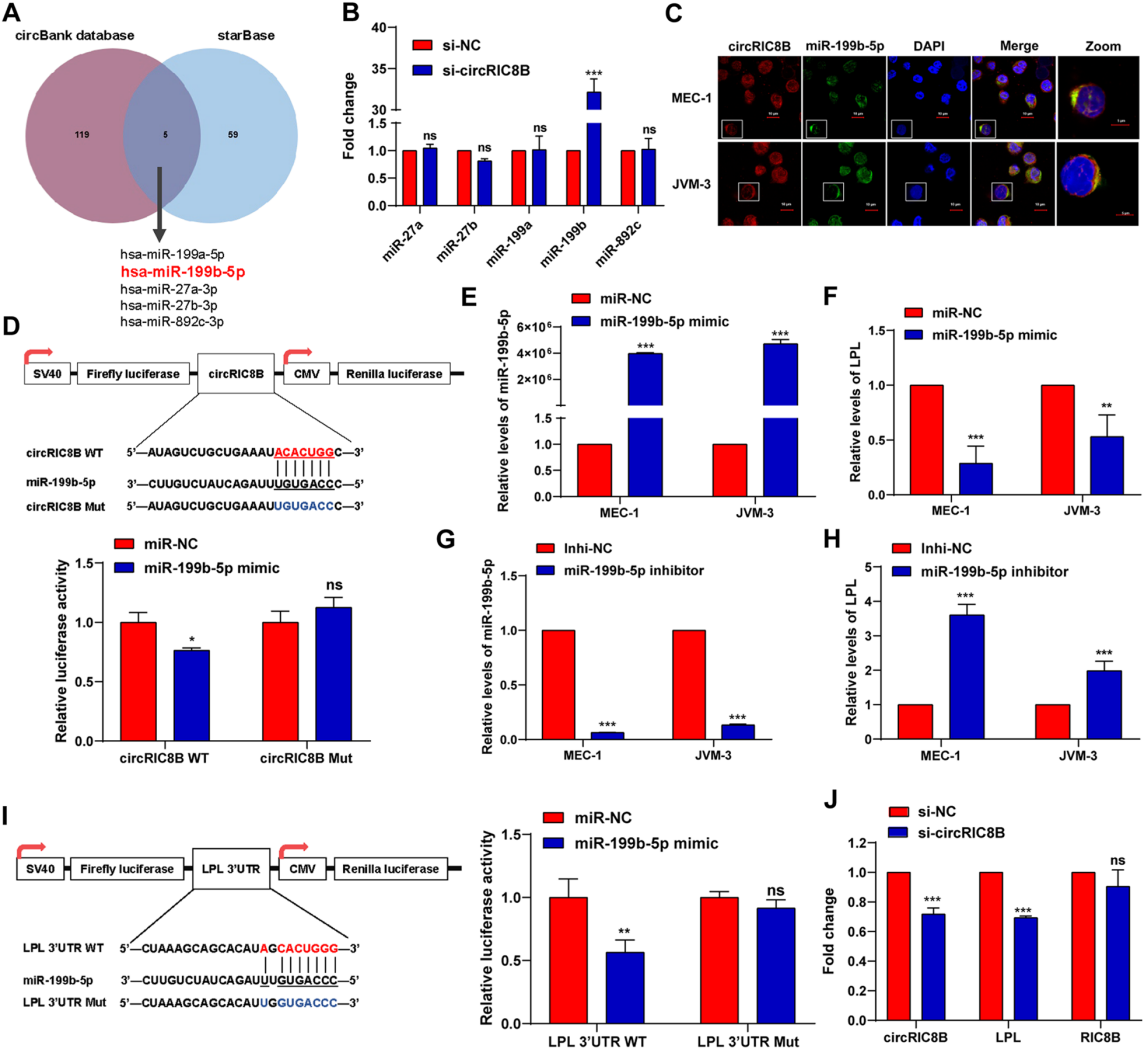
CircRIC8B serves as a miRNA sponge of miR-199b-5p to regulate LPL expression.** A** The overlapping of predicted circRIC8B target miRNAs and miRNAs targeting LPL. **B** miRNAs expression levels after silencing circRIC8B**. C** FISH assay for circRIC8B and miR-199b-5p. Scale bar, 5 μm. **D** Upper: Putative binding sites of miR-199b-5p concerning circRIC8B. Lower: Luciferase activity of pLG3- circRIC8B in HEK293T cells after co-transfection with miR-199b-5p. Expression levels of **E** miR-199b-5p and **F** LPL. Expression levels of **G** miR-199b-5p and **H** LPL were measured by qRT-PCR analysis after transfecting with miR-199b-5p inhibitor. **I** Left: Diagram of putative miR-199b-5p binding sites in the 3′-UTR of LPL. Right: Relative activities of luciferase reporters containing LPL 3′-UTR variants co-transfected with miR-199b-5p or negative control mimics in HEK293T cells. **J** Relative RNA levels after silencing circRIC8B. WT, wild type; Mut, Mutated; Data are shown as means ± SD (n = 3, ns, no significant, ^*^P < 0.05, ^**^P < 0.01, ^***^P < 0.001)

Given that circRIC8B was distributed in the cytoplasm and positively correlated with LPL mRNA, we investigated whether circRIC8B acted as a microRNAs (miRNAs) sponge in CLL cells. RNA immunoprecipitation (RIP) assay showed that circRIC8B was specifically enriched against Argonaute-2 (AGO2) (Additional file 8: Fig S4A). Then, we used circBank to predict miRNAs that bind circRIC8B and starBase to predict miRNAs that target LPL mRNA. The results showed that five miRNAs, including miR-199a/b, miR-27a/b, and miR-892c, overlapped (Fig. 6A). Notably, miR-199b-5p was the only one that was upregulated after transfection with circRIC8B siRNA (Fig. 6B). The FISH analysis confirmed the location of circRIC8B and miR-199b-5p mainly in the cytoplasm of CLL cell lines MEC-1 and JVM3, and importantly, they were co-localized (Fig. 6C). Dual-luciferase reporter assays were then performed. Compared with that in cells transfected with the mutant sequence, the luciferase reporter activity was significantly decreased by the miR-199b-5p mimics in cells transfected with the wild-type sequence (Fig. 6D). However, no significant change was observed when transfected with miR-199a, miR-27a/b, or miR-892c mimics (Additional file 8: Fig S4B). The change in LPL mRNA expression was verified after transfecting CLL cell lines with the miR-199b-5p mimic and inhibitor. LPL mRNA was downregulated with an increased level of miR-199b-5p and upregulated with a decreased miR-199b-5p (Fig. 6E–H). Luciferase reporter activity further confirmed the binding of miR-199b-5p and LPL mRNA (Fig. 6I). Additionally, LPL mRNA levels were significantly decreased after silencing circRIC8B (Fig. 6J). Collectively, our results provided evidence that circRIC8B can directly bind to miR-199b-5p and regulate the expression of LPL mRNA in CLL cells.

### CircRIC8B functions through the miR-199b-5p/LPL axis in CLL

**Fig. 7:**
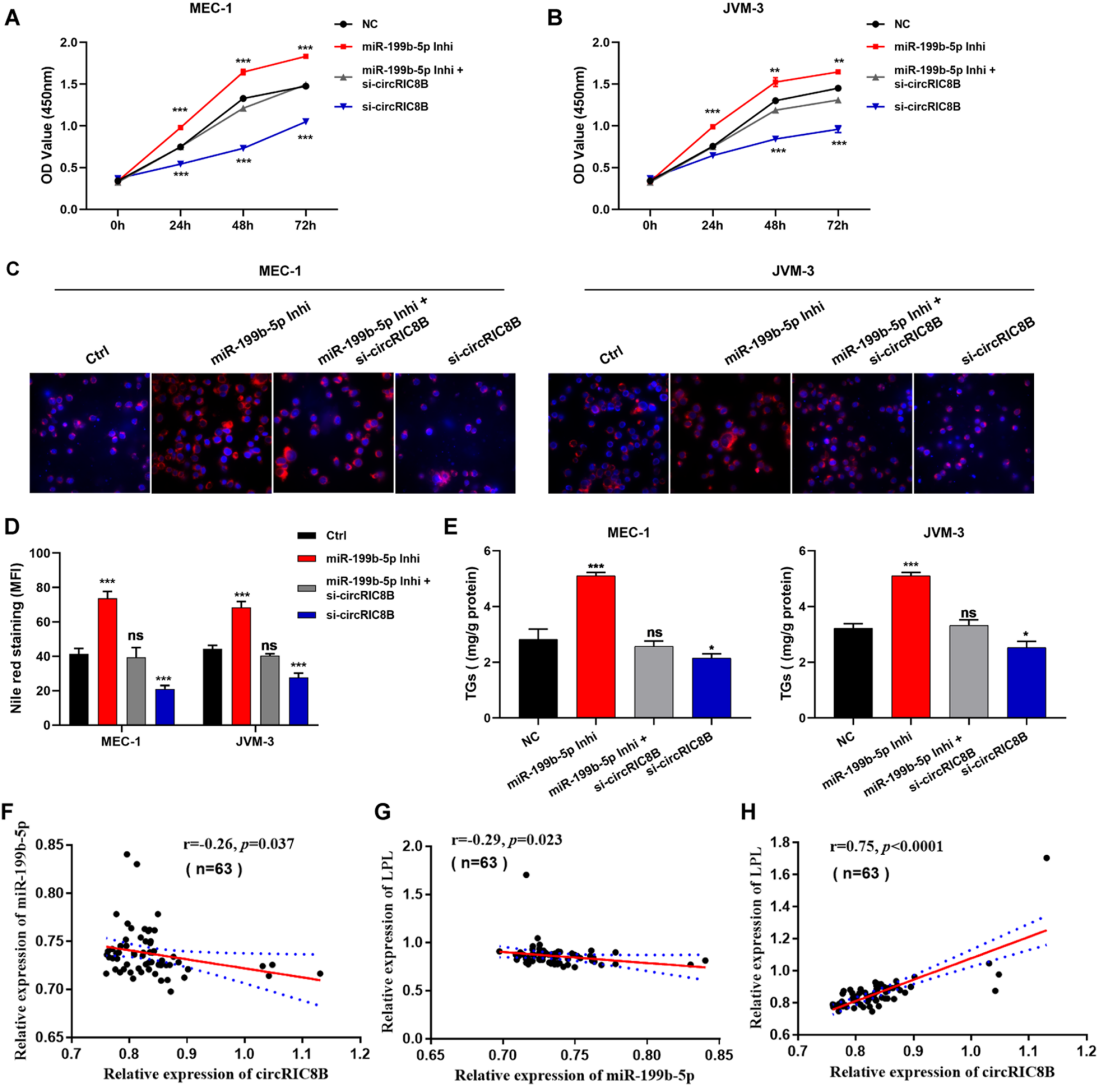
CircRIC8B involved in the progression of CLL by sponging miR-199b-5p. MEC-1 and JVM-3 were transfected with si-circRIC8B and/or miR-199b-5p inhibitors. **A**, **B** Cell proliferation was determined by CCK-8 assay. Lipid accumulation was determined by **C** Nile red staining and quantitatively analysed by **D** MFI. Scale bar, 10 μm. **E** TG contents. **F–H** Correlation analysis between circRIC8B, miR-199b-5p, and LPL expression levels in 63 CLL samples determined by qRT-PCR. Data are shown as means ± SD (n = 3, ns, no significant, ^*^P < 0.05, ^**^P < 0.01, ^***^P < 0.001)

We then verified whether circRIC8B induced CLL progression through the circRIC8B/miR-199b-5p/LPL axis. MEC-1 and JVM-3 were transfected with circRIC8B siRNA and/or miR-199b-5p inhibitor, and CCK-8 assays showed that circRIC8B knockdown significantly suppressed cell proliferation, whereas miR-199b-5p inhibitor induced proliferation. Transfection with miR-199b-5p inhibitor reversed the decrease of cell viability (Fig. 7A, B) and the reduction of lipid accumulation caused by circRIC8B knockdown in CLL cell lines (Fig. 7C, D). As expected, the changes in TG content showed the same results as the Nile red staining assays (Fig. 7E). Next, we investigated the expression and correlation of circRIC8B, miR-199b-5p, and LPL mRNA using PBMC samples from 63 CLL patients. Pearson correlation analysis indicated that miR-199b-5p was negatively correlated with circRIC8B and LPL mRNA, whereas circRIC8B levels were positively correlated with LPL mRNA (Fig. 7F–H). Together, these results verified the function of circRIC8B/miR-199b-5p/LPL mRNA axis in CLL.

### CircRIC8B knockdown enhances the sensitivity of CLL cell lines to ezetimibe, which inhibits cell viability and LPL mRNA expression

**Fig. 8:**
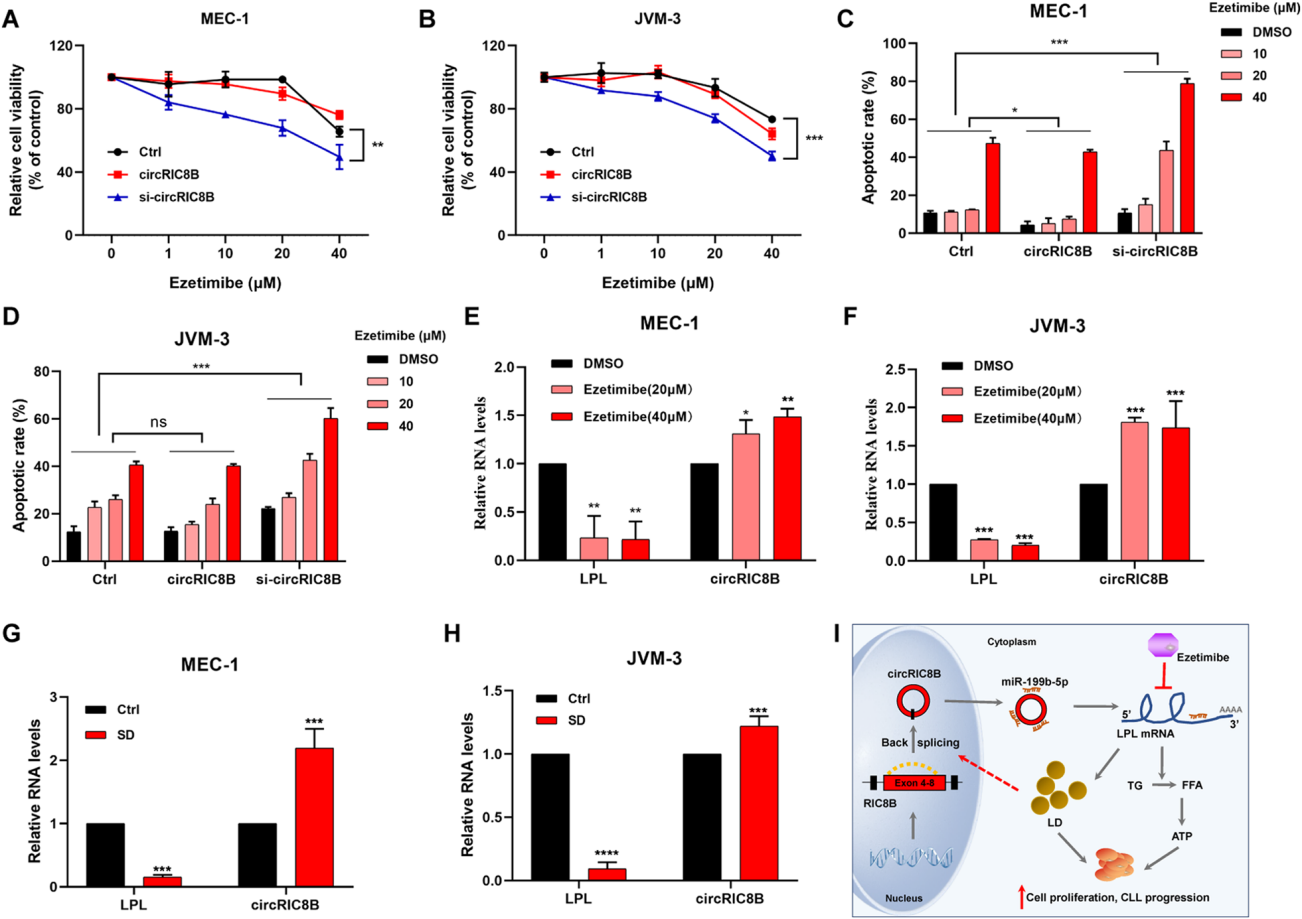
Cholesterol absorption inhibitor inhibits cell viability of CLL cell lines. **A**, **B** CCK-8 assay and **C, D** apoptotic assay of circRIC8B-knockdown/overexpression and control cell lines of CLL with ezetimibe treatment at the indicated concentrations for 48 h (n = 3). **E**, **F** Expression of LPL mRNA and circRIC8B in cells treated with ezetimibe. **G**, **H** Expression change of LPL and circRIC8B after SD treatment. SD, serum deprivation. **I** Schematic diagram illustrated the circRIC8B regulating pathway in CLL and the possible mechanism underlying ezetimibe. circRIC8B promoted CLL progression by sponging miR-199b-5p and regulated LPL expression. Suppression of LPL by ezetimibe caused inversely induction of circRIC8B. Data are shown as means ± SD (n = 3, ns, no significant, ^*^P < 0.05, ^**^P < 0.01, ^***^P < 0.001)

CLL cell lines with ezetimibe and we observed cell growth inhibitory effects in a dose-dependent manner. Notably, the inhibitory effects were much more obvious in the stable cell lines transfected with sh-circRIC8B. However, there was no significant difference between the control groups and circRIC8B overexpression groups in ezetimibe sensitivity (Fig. 8A, B). A similar result was observed in the apoptotic analysis (Fig. 8C, B, Additional file 9: Fig S5). The expression levels of LPL mRNA and circRIC8B were determined to further explore the role of ezetimibe. Ezetimibe markedly induced the reduction of LPL, whereas led to the enhancement of circRIC8B (Fig. 8E, F). Serum deficiency could lead to a reduction in lipid synthesis and thus cause the downregulation of LPL mRNA. Consistently, our data confirmed that serum deprivation caused the same change in the expression of LPL and circRIC8B (Fig. 8G, H).

## Discussion

In order to meet the increased demand for energy and metabolites owing to the rapid proliferation and survival of tumor cells, metabolic reprogramming was subsequently driven [18, 19]. CLL is a disease with approximately 1% of tumor cells proliferating every day, more than commonly thought [20]. Although CLL cells maintain high levels of proliferation through metabolic changes, extensive studies have not clearly explained the underlying mechanism of driving genes in the progress [21]. CircRNA is one of the types of non-coding RNAs, the novel functions and molecular mechanisms of which in hematological malignancies have attracted researchers’ attention [22]. Meanwhile, circRNAs were reported to exert vital functions in the metabolism of multiple tumors [23, 24]. However, the metabolic profile and role of circRNAs in the regulation of CLL metabolism remain largely unclear.

We investigated the circRNA expression profile in PBMCs from 53 patients with CLL using whole transcriptome sequencing and analysed the circRNAs involved in metabolism pathways. A new circRNA termed circRIC8B was observed to be remarkably correlated with LPL and significantly associated with the poor prognosis of patients with CLL. In addition, circRIC8B was a key factor in lipid accumulation through the miR-199b-5p/LPL axis, resulting in significant changes in cellular lipid storage, thus supporting the proliferation of CLL cells (Fig. 8I). Multiple reports showed that ncRNAs, such as lncRNAs, miRNAs, and circRNAs, contribute to tumor metabolism [24]. For example, miR-199b-5p, recognized as a tumor-suppressive miRNA, is involved in the lipid metabolism of breast cancer [25]. We have been dedicated to the study of circRNAs in CLL and reported that plasma circ-RPL15 serves as a diagnostic and prognostic marker and mitochondrial genome-derived circRNA mc-COX2 functions as an oncogene in CLL [15, 26]. To date, only a few circRNAs have been verified and explored in CLL [27, 28]. In the present study, we focused on the circRNAs participated in the metabolism modulation. Dramatically, our findings revealed new clues for the role of circRNA in abnormal lipid metabolism in CLL.

The estimation of tumor metabolism has a critical prognostic value in the evaluation of patients’ responses to treatment [29]. Targeting specific metabolism genes has shown clinical significance in the elimination and management of tumors [30, 31]. Increased LDL-C levels are strongly associated with an increased risk of coronary artery disease and other atherosclerosis-related disorders [32, 33]. Ezetimibe is a cholesterol absorption inhibitor that is reported to be an effective and generally well-tolerated option to reduce LDL-C concentration and confirmed to be effective and generally well-tolerated in patients [34]. The addition of ezetimibe to statin therapy helps to reduce LDL-C concentration and provides additional cardiovascular benefits [35]. Recent studies suggest there is a high incidence of elevated LDL levels in CLL patients. Thus, lipid lowering therapy may benefits CLL. Statin drugs reduce low-density lipoprotein (LDL)-cholesterol (LDL-C) and cardiovascular risk. Ezetimibe may be used to supplement statin therapy, or used alone in cases of statin intolerance [36, 37]. As LPL was the target gene of circRIC8B, which was positively correlated with LDL-C, and contributed to lipid accumulation, ezetimibe was then applied to assess its antitumor function. As expected, ezetimibe showed effective inhibitory effects on CLL cells by reducing the levels of LPL. Knockdown of circRIC8B significantly suppressed the lipid accumulation and proliferation of CLL cells. What’s more, decreased expression of circRIC8B also enhanced their sensitivity to ezetimibe, which indicated that higher levels of circRIC8B may be the factor that blocked the effectiveness of ezetimibe. Meanwhile, the levels of circRIC8B were upregulated with ezetimibe treatment, which may be explained by a compensatory regulation underlying some other signaling pathways while this needs to be further illustrated in future study. These results indicated that circRIC8B contributes to the maintenance of CLL cells during the inhibition of lipid metabolism. Ezetimibe, which exerts potential anti-oncogenic effect may be implicated in lipid metabolism through diverse regulatory mechanisms from circRIC8B. Our findings indicated that targeting circRIC8B with the addition of metabolic inhibitors may be a potential therapeutic strategy for CLL.

## Conclusions

To our knowledge, this is the first study to thoroughly investigate the relationship between circRNAs and metabolism in hematological diseases with the largest cohort. Our data provides additional evidence for the functions of circRNA in lipid metabolism and CLL pathogenesis, which may broaden the research horizons.

## Supplementary Information

**Additional file 1**: **Table S1**. Primer sequences used in qRT-PCR and PCR analysis

**Additional file 2**: **Table S2**. Metabolic score of 53 CLL patients

**Additional file 3**: **Table S3**. Genes that positively correlated with circRIC8B (R>0.5).

**Additional file 4**: **Figure S1.** Expression and clinical significance of LPL. A The association between LPL expression level and overall survival time was analysed by Kaplan–Meier plot. Log-rank tests were used to determine the statistical significance. B Comparison of TTT among patients with high or low LPL levels. The expression of LPL was significantly higher in patients with IGHV unmutated status than in those with mutations with C our data and D database. *, p < 0.05, **, p < 0.01, ***, p < 0.001.

**Additional file 5**: **Figure S2.** Relative levels of circRIC8B. A The expression of circRIC8B in CD19+ PBMCs and other cells from CLL patients (n=10). B Relative expression of circRIC8B in CLL cell lines MEC-1 and JVM-3 and human B lymphocyte cell line GM12878. Data are shown as means ± SD (n = 3, *P < 0.05, ***P < 0.001).

**Additional file 6**: **Figure S3.** Validation of overexpression and knockdown specificality. A The whole sequence of circRIC8B (710bp). B Sanger sequence of PCR products. C, D The expression of RIC8B detected by qRT-PCR and western blotting after circRIC8B overexpression or knockdown. Data are shown as means ± SD (n = 3, ns, no significant).

**Additional file 7**: **Figure S4.** circRIC8B acts as a miRNA sponge. A Levels of circRIC8B detected by qRT-PCR after RIP for AGO2 in CLL cells. B Luciferase activity of pLG3–circRIC8B in HEK293T cells after co-transfection with miR-199a-5p, miR-27a/b-3p, and miR-892c-3p mimics. Data are shown as means ± SD (n = 3, ns, no significant, *P < 0.05, **P < 0.01, ***P < 0.001).

**Additional file 8**: **Figure S5.** Results acquired by flow cytometry Apoptotic assay of circRIC8B-knockdown/overexpression and control CLL cells with ezetimibe treatment at indicated concentrations for 48 h.

**Additional file 9**: **Table S4**. Differentially expressed metabolites analyzed by positive ion mode.

## Data Availability

The datasets generated and/or analysed during the current study are available in the National Center for Biotechnology Information Sequence Read Archive (SRA), PRJNA762572.

## Abbreviations

3’UTR: 3′ -Untranslated region
AGO2: Argonaute-2
β2-MG: β2-Microglobulin
CCK-8: Cell Counting Kit-8
ceRNA: Competing endogenous RNA
CircRNA: Circular RNA
CLL: Chronic Lymphocytic Leukemia
DAPI: 4,6-Diamidino-2-phenylindole
FBS: Fetal bovine serum
FFA: Free fatty acids
FISH: Fluorescence in situ hybridization
gDNA: Genomic DNA
GSEA: Gene set enrichment analysis
IGHV: Immunoglobulin heavy-chain variable region
LC–MS/MS: Liquid chromatography with tandem mass spectrometry
LDL-C: Low-density lipoprotein cholesterol
LPL: Lipoprotein lipase
MFI: Mean fluorescence intensity
miRNA: MicroRNA
ncRNA: Non-coding RNA
OPLS-DA: Orthogonal partial least squares discriminant analysis
OS: Overall survival
PBMC: Peripheral blood mononuclear cell
PCA: Principal component analysis
qRT-PCR: Real-time quantitative polymerase chain reaction
RIP RNA: Immunoprecipitation
RNA-seq: RNA sequencing
RNAi: RNA interfere
ROC curve: Receiver operating characteristic curve
SD: Serum deprivation
siRNA: Small interfering RNA
STR: Short tandem repeat
TCA: Tricarboxylic acid
TCGA: The Cancer Genome Atlas
TG: Triglyceride
TTT: Time to first treatment
