# Supplementary figures and images for: CircRIC8B regulates the lipid metabolism of chronic lymphocytic leukemia through miR199b-5p/LPL axis
Source: Exp Hematol Oncol. 2022 Sep 5;11:51. doi: 10.1186/s40164-022-00302-0 (PMC9442988; doi:10.1186/s40164-022-00302-0)

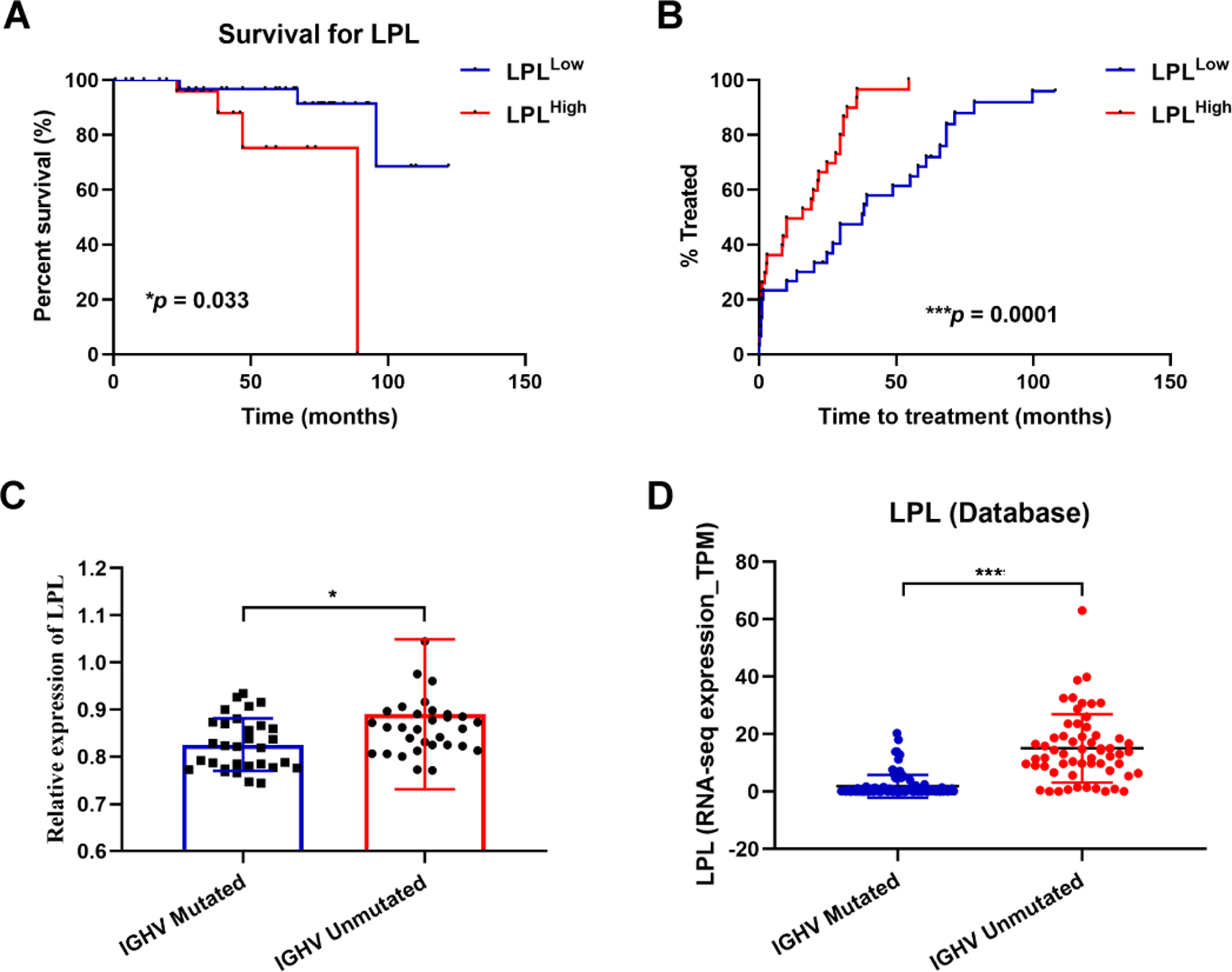

Supplement: Supplementary file 4 — Additional file 4: Figure S1. Expression and clinical significance of LPL. A The association between LPL expression level and overall survival time was analysed by Kaplan–Meier plot. Log-rank tests were used to determine the statistical significance. B Comparison of TTT among patients with high or low LPL levels. The expression of LPL was significantly higher in patients with IGHV unmutated status than in those with mutations with C our data and D database. *, p < 0.05, **, p < 0.01, ***, p < 0.001. [file 40164_2022_302_MOESM1_ESM.tif]

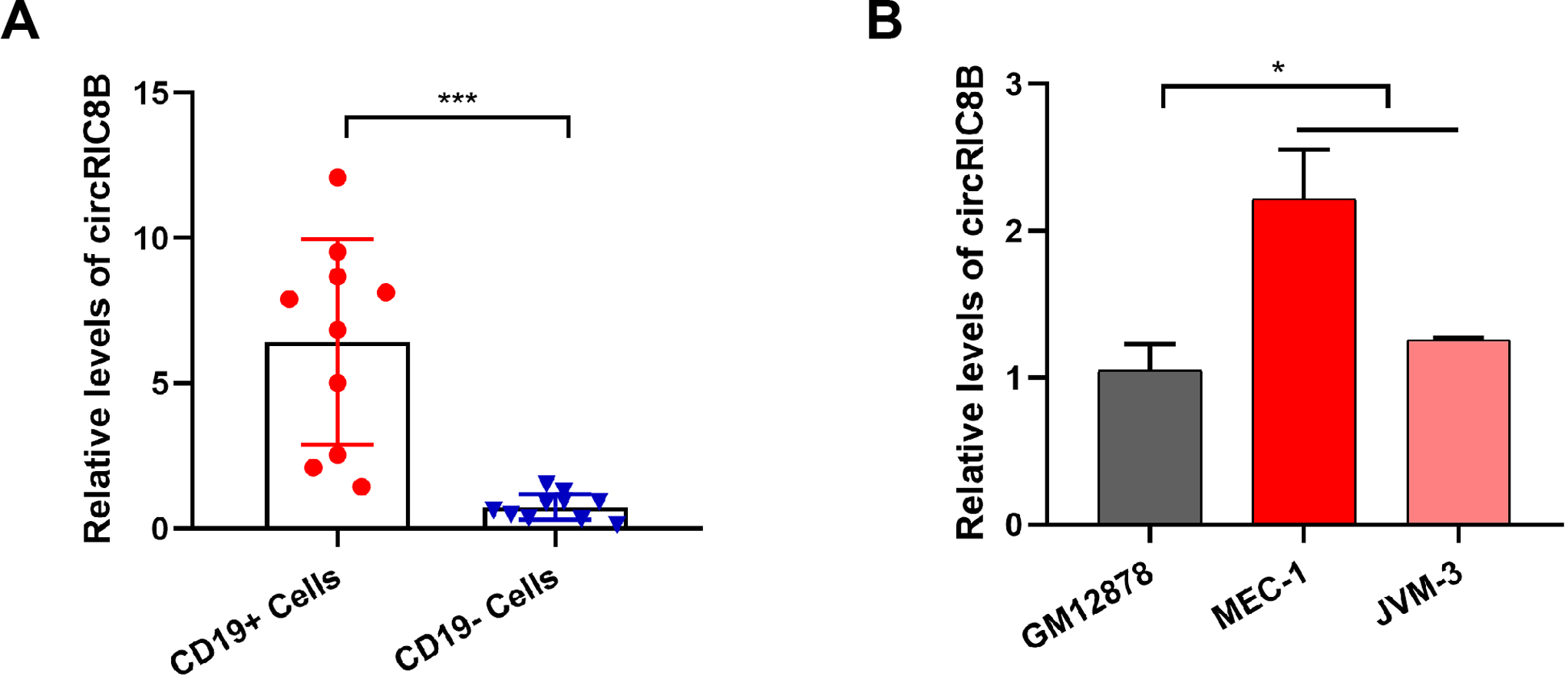

Supplement: Supplementary file 5 — Additional file 5: Figure S2. Relative levels of circRIC8B. A The expression of circRIC8B in CD19+ PBMCs and other cells from CLL patients (n=10). B Relative expression of circRIC8B in CLL cell lines MEC-1 and JVM-3 and human B lymphocyte cell line GM12878. Data are shown as means ± SD (n = 3, *P < 0.05, ***P < 0.001). [file 40164_2022_302_MOESM2_ESM.tif]

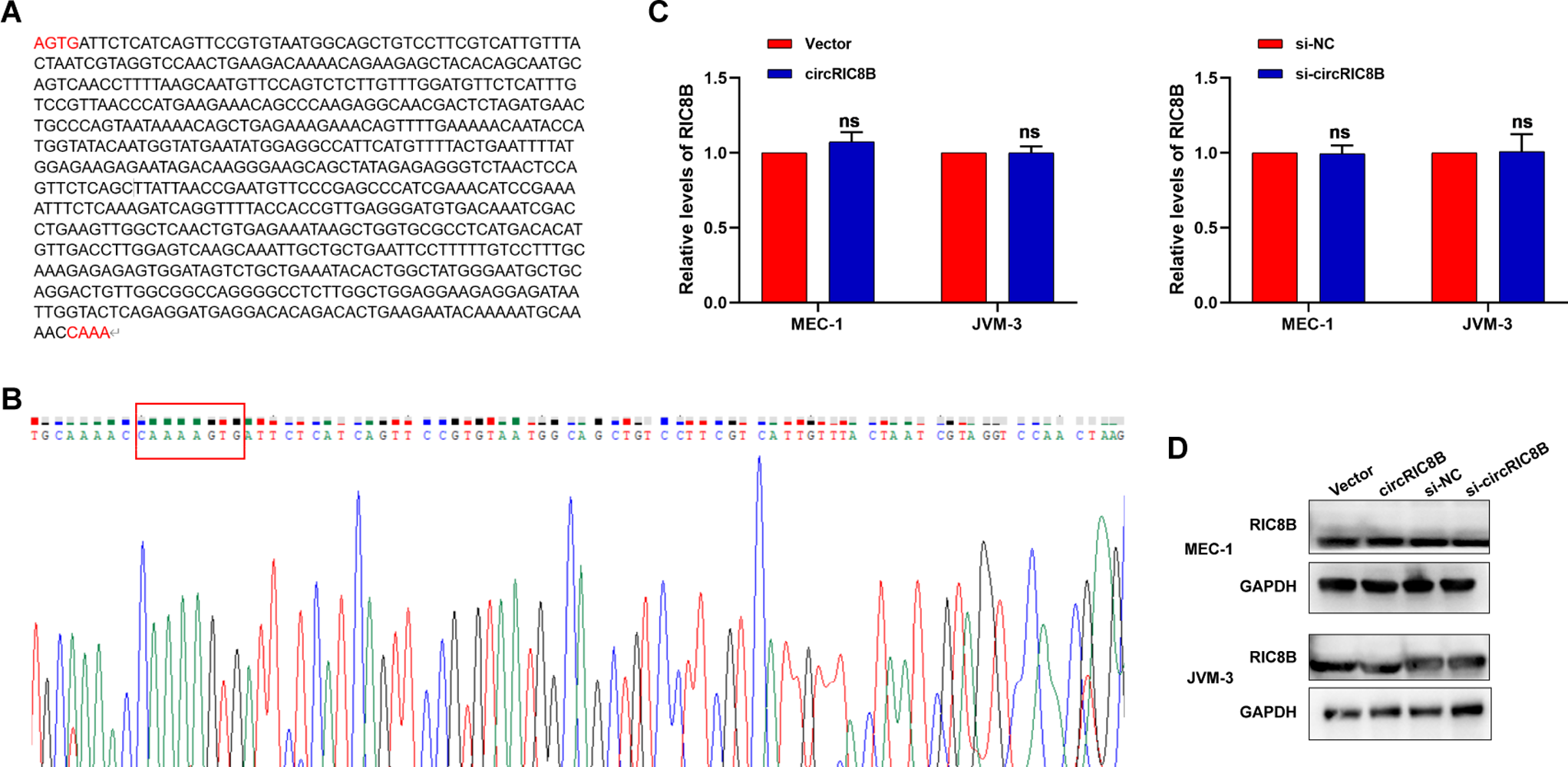

Supplement: Supplementary file 6 — Additional file 6: Figure S3. Validation of overexpression and knockdown specificality. A The whole sequence of circRIC8B (710bp). B Sanger sequence of PCR products. C, D The expression of RIC8B detected by qRT-PCR and western blotting after circRIC8B overexpression or knockdown. Data are shown as means ± SD (n = 3, ns, no significant). [file 40164_2022_302_MOESM3_ESM.tif]

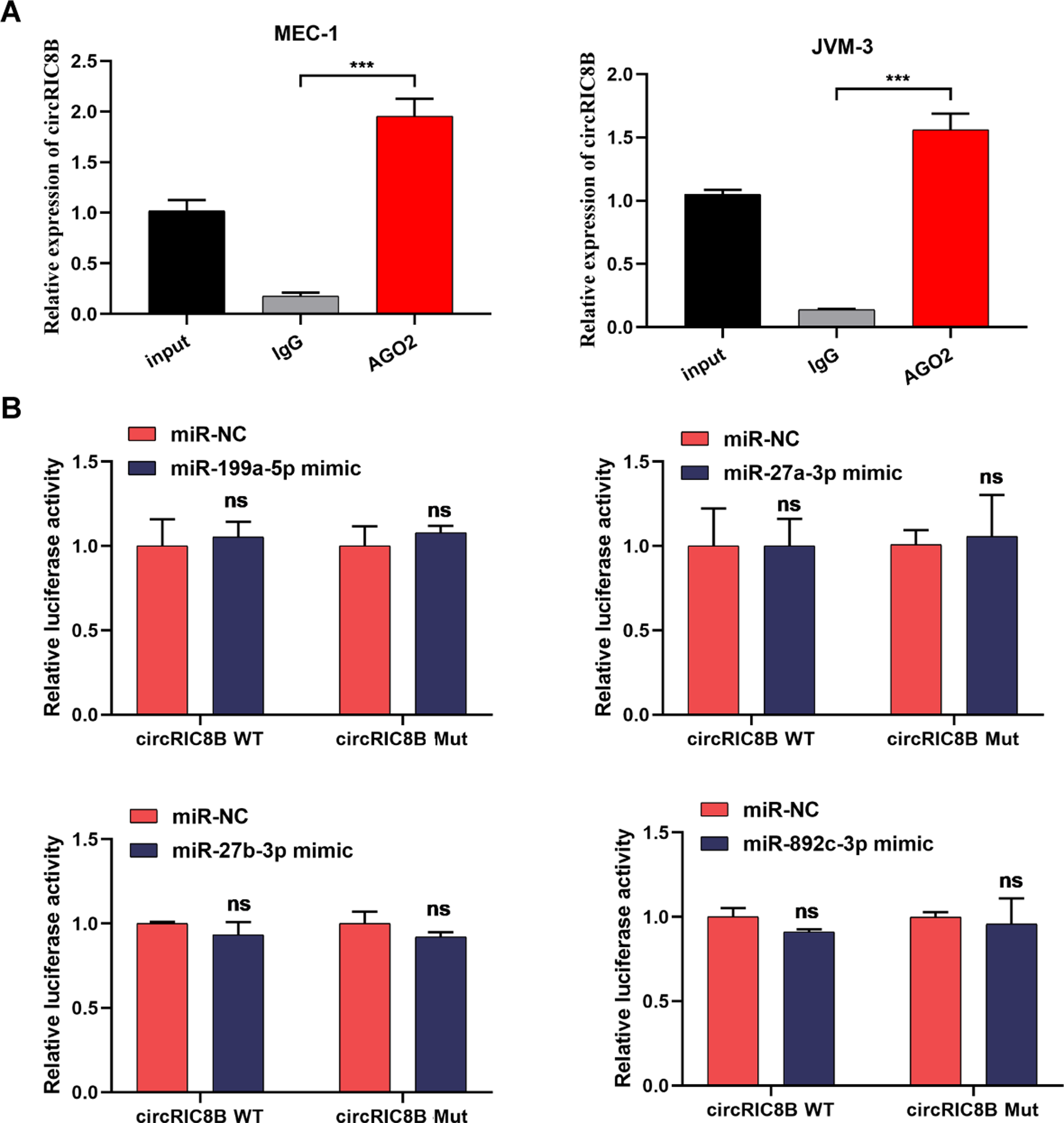

Supplement: Supplementary file 7 — Additional file 7: Figure S4. circRIC8B acts as a miRNA sponge. A Levels of circRIC8B detected by qRT-PCR after RIP for AGO2 in CLL cells. B Luciferase activity of pLG3–circRIC8B in HEK293T cells after co-transfection with miR-199a-5p, miR-27a/b-3p, and miR-892c-3p mimics. Data are shown as means ± SD (n = 3, ns, no significant, *P < 0.05, **P < 0.01, ***P < 0.001). [file 40164_2022_302_MOESM4_ESM.tif]

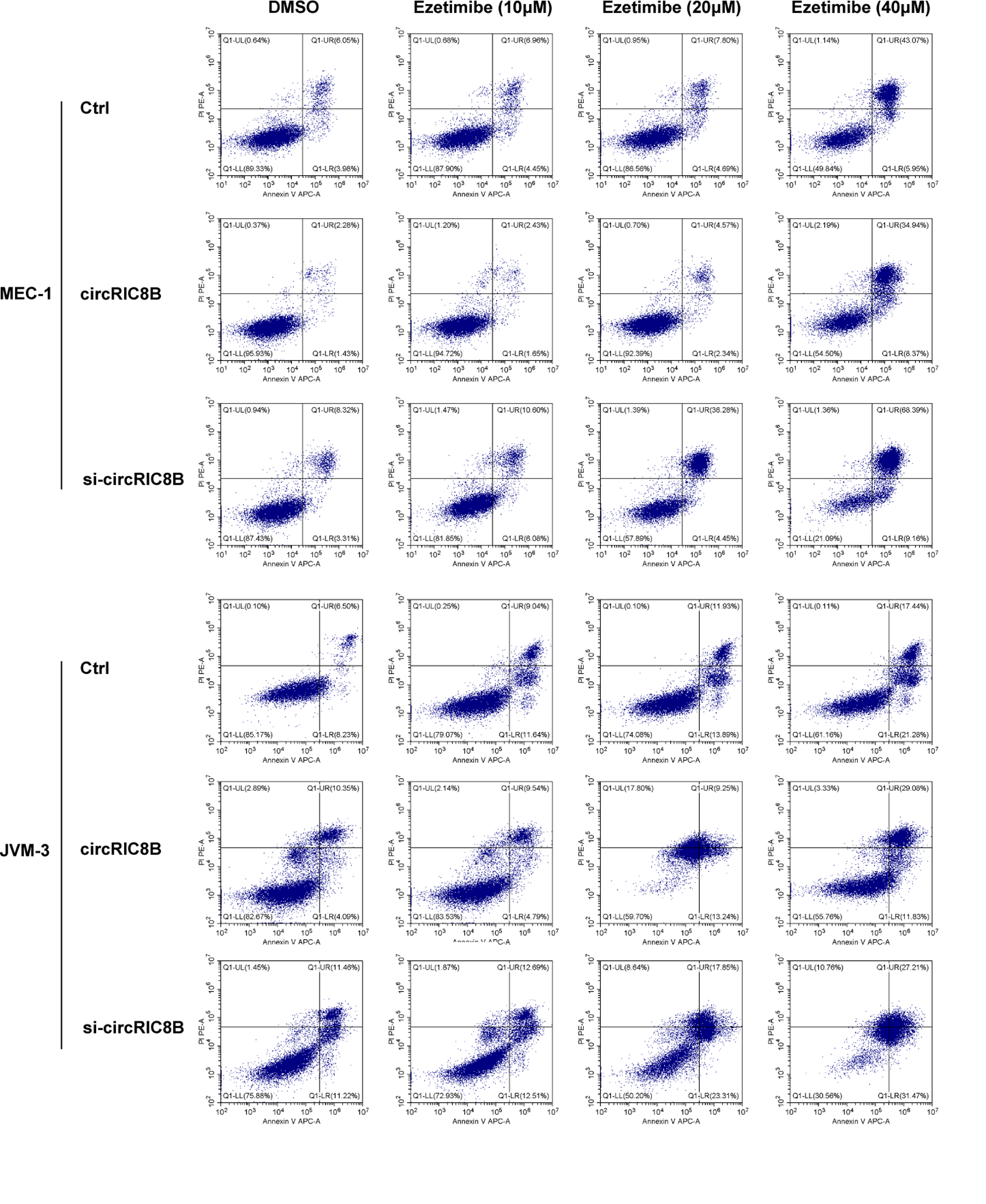

Supplement: Supplementary file 8 — Additional file 8: Figure S5. Results acquired by flow cytometry Apoptotic assay of circRIC8B-knockdown/overexpression and control CLL cells with ezetimibe treatment at indicated concentrations for 48 h. [file 40164_2022_302_MOESM5_ESM.tif]
